# Mental health needs and services in the West Bank, Palestine

**DOI:** 10.1186/s13033-016-0056-8

**Published:** 2016-03-15

**Authors:** Mohammad Marie, Ben Hannigan, Aled Jones

**Affiliations:** College of Medicine and Health Sciences, Annajah National University, 7, Rafedia, Nablus, West Bank, Palestine; School of Healthcare Sciences, College of Biomedical and Life Sciences, Cardiff University, Eastgate House, 35-43 Newport Road, Cardiff, CF24 0AB UK

**Keywords:** Conflict, Mental health system, Health policy, Palestine, Resources, Resilience, Community mental health nursing

## Abstract

**Background:**

Palestine is a low income country with scarce resources, which is seeking independence. This paper discusses the high levels of mental health need found amongst Palestinian people, and examines services, education and research in this area with particular attention paid to the West Bank.

**Methods:**

CINAHL, PubMed, and Science Direct were used to search for materials.

**Results and conclusion:**

Evidence from this review is that there is a necessity to increase the availability and quality of mental health care. Mental health policy and services in Palestine need development in order to better meet the needs of service users and professionals. It is essential to raise awareness of mental health and increase the integration of mental health services with other areas of health care. Civilians need their basic human needs met, including having freedom of movement and seeing an end to the occupation. There is a need to enhance the resilience and capacity of community mental health teams. There is a need to increase resources and offer more support, up-to-date training and supervision to mental health teams.

## Background

The history of Palestine is marked by conflict. This challenging political context has exerted effects on Palestinian mental wellbeing and lifestyles. The 1948 War between Arabic countries and Israel was considered, by the Palestinians, as the beginning of the ‘Catastrophe’, known in the Arabic language as ‘Nakba’. Around three quarters of the Palestinian people were displaced or fled due to the conflict and were then considered refugees by the United Nations [1]. People lost their lives. Villages, homes and lands were lost in addition to people experiencing trauma, and feeling defeated [2]. The 1967 War had an additional negative effect on Palestinian wellbeing and on people’s daily lives. More Palestinians ended up living in an unstable environment or facing dramatic changes. Many experienced persecution, deprivation, discrimination and injustice [2].

In 1987, the first uprising, known as the Intifada, started in the Gaza Strip and the West Bank. Israeli punishment practices were used to curtail the Intifada [2]. In 1993, a peace process started between Israel and the Palestine Liberation Organization (PLO), later renamed the Palestinian Authority [1]. The Palestinian Authority started to manage many services in the occupied Gaza Strip and West Bank, including the health service [3]. In 2000, Israeli punishment measures and practices were again put in place, this time to discontinue the second Intifada [2].

In 2002, Israel started to build a physical barrier, with part of it placed between the Palestinians’ cities and villages. Israel called it a ‘fence’ [4] and Palestinians called it a ‘Separation Wall’. Currently this is nearly 300 miles long and 8 m (25 feet) in height. According to Abu ziada [5], this physical barrier has had a negative impact on the daily activities of most of the Palestinian people. In addition, the new changes increased the percentage of Palestinian people who are in need, especially farmers in poverty. The ‘Wall’ or ‘fence’ has increased the percentage of landless people. All in all, these new changes to the land were judged to have had a negative effect on the psychosocial needs of the people. Similar findings will be discussed in later sections that discuss the effect of conflict on the population.

In 2006, the Palestinian government experienced a boycott by most of the world’s influential countries, including Israel. This led to a significant lack of economic support and international aid. This had a negative effect on the running of services for civilians; health services were one of those areas most affected [3]. In 2008, 2012 and 2014, there was a prolonged siege involving movement restrictions on food and individuals especially in the Gaza Strip. Israel conducted another three military operations include tank shelling and aircraft bombing. According to Amnesty International reports, war crimes were conducted against Palestinians. As a result, more Palestinians experienced violation to their human rights, loss of life, harm and home destruction. More civilians experienced challenges to their mental well-being [6]. Most of the people in the West Bank were gravely concerned about their relatives in the Gaza Strip during the military operation [7].

In summary, the above history of occupation with political conflict caused major challenges to the Palestinian people. According to Afana et al. [8] historical events have made around one-third of Palestinians in need of mental health interventions which makes mental ill-health one of the largest but least acknowledged of all health problems. These events imprinted this conflict on the collective consciousness of the Palestinian state in a negative way [3]. This raises questions about the mental health needs of Palestinians who live in these chronic conditions as well as questions about the increased demands on mental health services. The following sections explain the developmental challenges on the mental health services in the surrounding countries or region.

### Development of mental health care in the Arabic region

In the Arab states, religion has played a key role in many aspects of people’s lifestyles and in political issues [9, 10]. The Islamic model of health care has been influenced by the cultural context, and is seen as inseparable from Islamic values [11]. For example, the most significant nurse in the Islamic history and Arabic culture was Rufaidah Al-Asalmiya (570-632 AD) [12]. The history of mental health services in the Arab countries goes back to the “golden” periods of Islamic civilization. Baghdad in Iraq had many mental hospitals established in the year 705 AD [13]. Muslim scientists established during the 10th century some of the basics which are practised nowadays [14].

However, mental health services and research in the Arabic region nowadays do not command such a leading position in science as they previously did. There is a near absence of effective mental health research, and mental health services are grossly underdeveloped, with stigma attached to those experiencing mental illness. The developmental challenges of mental health care vary from country to country influenced by their income. In addition, other factors have determined the efficacy of mental health services including political decisions, social factors and the specific diversity of cultures. The Arab countries have tried to develop their model of care and mental health services. They are trying to improve research in the mental health field and to train higher numbers of qualified professionals. They are also moving toward deinstitutionalization models and are trying to become independent and autonomous by suggesting more suitable ways of caring within the modern Muslim culture [15].

There are also specific cultural and contextual influences on health care in Palestine. For example, family cohesion and support are important aspects in the lives of patients and the family needs to be included in significant decisions [16]. The care plans created by Palestinian nurses are recognised as being affected by their cultural context and their environment [17].

## Aim

Against a background of conflict and turmoil, and in the broader context of the development of mental health care in the Arabic region, the aim of this review article is to examine the mental health needs of Palestinians and the availability of services, before making recommendations for policy, development and practice.

## Method

For the search strategy, databases were scanned broadly prior to refining the searches. CINAHL (Cumulative Index to Nursing and Allied Health Literature) and PubMed were searched. Key search words included, ‘Palestin*AND mental health’. Science Direct was also searched by using mental health AND screening AND Palestine. These words were also used to search in the Arabic language to identify articles indexed in Annajah University Journal for Research. Additional papers, which did not come to light in the electronic database search, were obtained via an examination of reference lists of published papers. This study included references about mental health in the West Bank only; studies outside the West Bank such as Gaza were excluded. The search identified 322 items. Duplicates were removed, and 43 relevant articles and reports were included in this review. For the critical analysis of empirical articles, the following aspects were considered: survey instrument used, aim, sample, data collection criteria, limitations or bias, key findings, ethical and procedural rigour. For the empirical studies with statistical methods the reliability and validity were taken into consideration and the framework of Rees [18] was consulted to help with these appraisals and critiques.

### Development of mental health services in Palestine

The health system in Palestine is in a transitional stage and facing specific contextual challenges linked with the occupation and political conflict [19, 20]. The health care system is complex and fragmented; basic public health and primary care are offered by four main facilities: the Palestinian Authority (Governmental), the United Nations (United Nation Relief and Work Agency for Palestinians), non-governmental organizations (NGOs), and private health care services such as pharmacies or clinics [20, 21].

After 1967, mental health services for Palestinians in West Bank and Gaza were managed by the Israeli government and became neglected and underdeveloped. Although mental health services needed to be prioritised, there was instead a significant lack of resources and trained professionals [13]. There was a need for effective secondary prevention, but a lack of infrastructure and the necessary qualified teams [22]. Since 1987, the NGOs have tried to help the Palestinians as much as possible and fill the gap in governmental services [22]. However, overall mental health services became stagnant at a time when conflicts and troubles meant they needed more development.

In 1993, after the Oslo peace agreement, the Palestinian Authority started to manage basic services including mental health care. It took over responsibility for an underdeveloped system, including a neglected system of mental health services and research [3, 23]. In 1996, community mental health services became an independent part of primary care and separated from psychiatric hospital management [3, 24].

In 2004, the need for mental health care among Palestinians became greater than before, especially during the Al-Aqsa Intifada. Many health services and clinics were affected negatively or destroyed due to the Israeli military practices [22]. Increasing numbers of Palestinians developed mental health difficulties [25]. According to Giacaman and Mikki [26] more patients became unable to reach the psychiatric hospital in Bethlehem due to movement restrictions on roads. A few governmental and nongovernmental institutions delivered psychosocial and community mental health services, but most were limited and depended on externally funded programmes. Moreover, the number of mental health professionals remained seriously limited [26].

In 2006, the Palestinian government experienced an acute lack of international funds and financial support. As a result, the health system was affected negatively; access to medicines and basic medical supplies sharply declined, and money was not available to pay salaries [27]. According to the World Health Organization (WHO) and Ministry of Health [28] community mental health has not been a priority in the financial budget of the Ministry of Health and is under-resourced. For example, the budget for mental health services consisted of 2 % of the whole budget of the Ministry of Health; and 73 % of the 2 % is spent on the psychiatric hospital. A lack of finances, management structure and human resources inhibited the quality of mental health services [28]. There is a recognised need to train new numbers of professionals and to strengthen the mental health workplace [29].

The movement restrictions prohibited more civilians from accessing support to meet their basic human needs. The WHO has been trying to develop the Palestinian health system. Consequently, WHO, in cooperation with the Palestinian Ministry of Health, has implemented a plan to develop a mental health system in Palestine including the West Bank [29]. This plan is based on moving away from hospital based in-patient services to community provision of care. As a result, WHO is building new community mental health centres in each city. The WHO has also trained increasing numbers of mental health professional teams, although the total number of mental health nurses continues to be very low. This is important, as nurses remain key to the development of health systems around the world where they remain, globally, the largest of the professional groups involved in the provision of care [29]. One example of an initiative which is developing nursing specifically is the WHO, in cooperation with Annajah National University in the West Bank and with the Islamic University in Gaza, establishing the first Master’s Degree in community mental health nursing in Palestine. This Master’s programme aims to prepare highly qualified mental health nurse graduates who can work in the community mental health centres [3, 24].

Currently, mental health services in the West Bank and East Jerusalem are based on the community provision of care. The main services are provided by the Ministry of Health but there are only 13 community mental health clinics or centres (CMHCs) in the West Bank, in addition to one psychiatric hospital in Bethlehem. In 2013, those outpatient facilities treated 87.7 new service users per 100,000 population. The service users treated in CMHCs were diagnosed with neurotic disorders (24.2 %), learning disability (mental retardation) (14.6 %), schizophrenia (12.2 %), epilepsy (10.7 %), affective disorders (9 %), other mental disorders (7.8 %), organic disorders (4.4 %), personality disorders (3.3 %) and substance abuse disorders (1.7 %) [30]. The quality and quantity of care needs improvement in these community mental health services [31].

There is a national steering committee for mental health which draws up mental health policy or plans [28]. There is a lack of evidence that service user associations are represented at this committee. Mental health policy in Palestine has also developed towards placing mental health services under primary care, rather than hospital, management [3, 24]. However, services have suffered through an overload of demand, lack of up-to-date medications and an ineffective management structure inside the community centres. Some mental health teams have been unable to share effectively in treatment plans or provide high quality care [32]. There is a lack of effective programmes in fighting the stigma toward mental illness such as through documentary films. There is lack of up-to-date training or continuous education, interventions, and research and there is a lack integration or connections between Palestinian mental health professionals and their international colleagues. There is a lack of integration between primary health care teams and community mental health services [20].

Mental disorders in Palestine remain underreported, under-resourced, under-treated, and mental health services underfunded. These services are unable to meet the burden of need. There is a severe lack of human and infrastructure resources, for example the total number of psychiatrists is 20 in the West Bank [20]. Each community mental health centre or clinic contains mostly one psychiatrist, psychologist or social worker in addition to one not well-trained or specialised mental health nurse [32]. The total number of nurses who work in community mental health workplaces in the West Bank is only 17, working with a total population of nearly three million [33]. A comparison can be made with Wales (UK), a country with a similarly sized population to the West Bank but with a total number of community mental health nurses of at least 600 [34]. In addition, the Palestinian nurses who work in these clinics or centres have been unable to provide mental health care properly and some of them work as receptionists or clerks due to the severe shortage of other employees or lack of training. There remains a need to develop the quality and quantity of mental health care in these community services [33].

In summary, mental health services have been mostly underdeveloped, under resourced, under researched and under supported [35]. The mental health system has been affected negatively by the political conflict and this is thought to increase the challenges facing health workers in their daily routines.

### Mental health of Palestinians

The findings in the below studies need to be considered with caution due to weaknesses in study design, such as the use of self-developed and unverified questionnaires and measurement tools that are in need of further validation. For example, Espie et al. [36] undertook a study to describe the occurrence and treatment of psychiatric disorders in the Palestinian populations of the Nablus district in the West Bank. From 2005 to 2008, 1369 patients were clinically assessed using a semi-structured interview based on DSM-IV-TR criteria. Among 1254 patients, 15.3 % reported depression, 17.3 % anxiety disorder (other than PTSD or acute stress disorder), and 23.2 % post-traumatic stress disorder [PTSD]. Among children ≤15 years old, factors significantly associated with PTSD included being witness to physical abuse or murder, receiving threats, and property destruction or loss.

Dimitry [37] systematically reviewed the literature on the mental health of children and adolescents living in areas of armed conflict in the Middle East which include Palestine. PubMed was searched and papers were identified using specific inclusion criteria. The main findings were that children and adolescents living in these conflict zones are exposed to high levels of traumatic experiences. Numbers of conflict-related traumatic experiences correlate positively with prevalence of mental, behavioural and emotional problems. Prevalence of post-traumatic stress disorder in Palestinian children and adolescents is estimated to be 23–70 %. These findings bring to light the pressing need to provide children and adolescents living in conflict areas with help.

Giacaman et al. [38] conducted a survey of Palestinian adolescents in school in order to investigate collective and individual exposures to violence and its negative effect on adolescents’ mental health. A representative sample of 3415 students of 10th and 11th grades from the Ramallah District of the West Bank participated in the survey. The primary independent variables were scales of individual and collective exposures to trauma/violence (ETV) by the Israeli military and settlers. Outcome measures were constructed and included a binary measure of depressive-like states, and somatic scales. The level of exposure to trauma/violence was very high. For example, 80 % had seen shootings, 28 % had seen a stranger killed, 11 % had seen a friend or neighbour killed, and 54 % of boys had experienced body searches. In addition, 10.4 % of the participants had a depressive like state, 14.1 % emotional difficulties and 10.3 % somatic disorders. The findings confirmed that both collective and individual ETV independently affected the mental health of participants. The results of this study emphasize the importance of the concept of collectively in analysing violent and traumatic contexts. In the case of Palestinian youth, a community centred social approach as opposed to an individualistic therapeutic one may better help in assisting youth in coping with the social suffering of war.

Madianos et al. [39] undertook a cross sectional quantitative study designed to investigate the lifetime and one-month prevalence of a major depression episode (MDE). The sample consisted of 916 adult Palestinians in the West Bank. The clinical examination used DSM-IV criteria for the detection of MDE. Data on sociodemographic, suicidal behaviour, previous help-seeking, medication use and exposure to trauma were also collected. The study found that the lifetime and 1 month prevalence of MDE was 24.3 and 10.6 %, respectively. The vast majority of males in this sample (85 %) and 69 % of females confirmed that they had experienced a serious traumatic event.

Ghrayeb et al. [40] conducted a descriptive study aimed to determine the prevalence of mental ill-related behaviour among 720 Palestinian adolescents in Tarqumia in the West Bank. Students aged 13–17 years in four public secondary and high schools anonymously completed the Arabic version of the international Global School-Based Health Survey. The findings revealed that 25.28 % had made suicide attempts and 24.58 % had suicide ideation. The prevalence of feeling lonely was reported by 25.28 % of male and female adolescents. Further mental health interventions are needed to reduce the trend of suicide and the escalating suicidal ideation among the most vulnerable populations.

Another study was conducted by Giacaman et al. [41] in five towns in the West Bank area, all of which had been exposed to military intervention from the Israeli army. Data were collected using two-stage cluster-sampling. The sample consisted of 153 households in Ramallah city, 154 in Nablus city, 148 in Bethlehem, 151 in Jenin and 155 in Tulkarim. Each town was divided into five strata then stratified random sampling of houses was undertaken and mostly equally divided between both genders of the household. The researchers used a self-developed questionnaire that consisted of ten items measuring housing, financial and health related issues. Interviews were conducted with adult household members, with the sample of respondents almost equally divided between men and women. One of the health scale questions was: “did you or anyone in your home have psychological distress?’’ The findings showed that households including people with the highest percentages of distress were in Ramallah city (93 %), then Tulkarim (91 %), Jenin (89 %), Bethlehem (87 %) and Nablus city (71 %). Interviews with respondents revealed that psychological distress was associated with witnessing unpleasant events. For example, responders reported high psychological distress at home such as: uncontrollable fear, hopelessness, fatigue, depression, sleeplessness, shaking episodes, and uncontrolled crying episodes or enuresis in children.

A quantitative and descriptive analytic study was carried out to determine the most significant psychological problems that affected Annajah National University students during the Alaqsa Intifada [42]. The random stratified sample consisted of 586 students, of whom 566 responded. The researcher used a self-developed questionnaire that consisted of three parts. The first part included a section to gather demographic data. The second part consisted of a psychological problems questionnaire that contained 73 items. These items were developed by the researcher and were also reviewed by seven local mental health experts. For example, one of the questions asked the respondents if they felt frustrated and stressed continuously due to the conflict. The third part contained open questions that asked the student to write three positive things. Most of the students complained about psychological disorders. Moreover the study showed that participants experienced a variety of symptoms such as: anxiety, fear, frustration, lack of feelings of security, psychological stress, hopelessness, helplessness and fatigue.

To investigate the mental health of women, a quantitative study by Assaf and sha’th [43] was undertaken in North Palestine Districts to determine the effects of Israeli army practices on Palestinian women’s stress during the Alaqsa Intifada. Women are considered the core of Palestinian society so if there is any pressure on society then that will be exerted on the most important person in the family, which is the mother. This random sample study included 900 women from different education levels, social classes and districts. The researcher used a self-developed questionnaire that consisted of 77 items. The study found that the average degree of psychological, economic, and social pressure among the participants was high (69 %). This meant that most of the women experienced a high degree of suffering and complained of collective distress due to the conflict. The participants also reported that the sense of solidarity between Palestinians was increased during adversities.

Finally, the mental health of Palestinian children may be affected even more than the mental health of adults. Arafat and Boothby [44] carried out a study in which they found that 93 % of children had experienced being or feeling threatened, a loss or a lack of security, and fear. The fear was not about themselves alone, but also about their families and friends. The team developed three sets of questions, targeting children, parents and teachers respectively. The questionnaires were specifically developed for use in focus group discussions. Parents also reported many psychological symptoms in their children such as nightmares, enuresis, and high levels of aggressive behaviours, hyperactivity, low attention and concentration.

Overall, there is a lack of qualitative mental health studies and one shared limitation for the above quantitative studies is that they need more strategies to enhance the credibility and validity of the findings. For example, one-off self-designed questionnaires were used and reviewed by the local experts. Alpha Cronbach equation tested most of the reliability of the studies for internal consistency which was found to be more than good. From the above studies, it seems quantitative studies are more common in Palestine as they are less time consuming or costly than large scale qualitative studies. It also appears that Palestinian researchers are not working collaboratively to develop Palestinian-specific measures by building on and developing each other’s work. All in all, these studies give us insight, albeit limited at times, about the mental health of Palestinian people in the absence of more accurately representative surveys in this field [23]. In particular it seems that there is a lack of accumulation of knowledge across multiple studies especially in the area of mental health screening. The studies were also conducted during the Intifada period, so are more representative of mental health status when there are high levels of violence in the surrounding environment. These quantitative studies may explain the extent to which Palestinians live under chronic intense pressures, but qualitative studies are still needed to be able to develop these findings further.

## Discussion and conclusion

Given the extremes of war and deprivation experienced in Palestine over the last 70 years or so there is an immediate need to develop capability among mental health professionals and capacity within the service. In particular, there is a significant need to develop mental health services by strengthening and empowering the mental health staff members’ capabilities and resilience [7]. In addition, awareness needs to be raised about the stigma of mental ill-health, and greater integration of mental health within primary care services is required. Efforts need to be made to improve supervision systems and the capacity of mental health teams, and to increase the availability and quality of care [45]. There is a general lack of awareness of mental illness and a need to tackle the misperceptions and stigmatising attitudes of nurses and other health professionals [8].

There is also a priority to listen to health professionals’ needs, and to support them in order to enable their resilience and to help them cope with their daily challenges [33]. Many of the international funders connect a biomedical approach to mental health treatment and their funding. For example, they focus on PTSD as an individual problem but without a focus on its social, political and cultural context. The Palestinian community has cohesiveness and most of the community is exposed to violence. The depressive-like effect will be found in individuals as part of cumulative collective exposure [38]. In other words, in Palestinian culture, trauma is mostly interpreted according to specific collective meanings more than individual experiences. Some international funders spend funds without basing these on need assessments related to the Palestinian context. For example, they advertise in the newspapers that their project will achieve some goals such as women’s civilian rights then the mental health agencies try to follow and work on these themes which are not necessarily the most urgent or most suitable priority for the whole population [3]. Mental health interventions need to be suitable for the cultural context, and to be based on public or community interventions [46].

In addition, a study undertaken by UNFPA and FAFO [47] showed that just 1 % of civilians would look for individual counselling, whereas most preferred to talk to close friends or to their family members. This study gives us an opportunity to focus on social support group models or improve the capacity of the whole community rather than focusing on approaches based on individual treatments. This includes developing the capacity or resilience of mental health teams who work in underprivileged workplaces with the addition of increasing life demands.

Mental health professionals who are part of the Palestinian population need to fulfil their basic needs as human beings such as maintaining their feelings of well-being. Bringing an end to the surrounding violence and movement restrictions promises to improve the mental health of civilians [48]. Overall, some international countries are dealing with chronic conflict in a superficial way, giving only urgent humanitarian food or aid. Furthermore, the priority need for civilians is to have their freedom such as to speak, travel and be protected. The continuously unstable environment is producing new mental health problems for civilians. The priority is to create an effective and supportive mental health system and stop the occupation as a primary prevention [22]. Palestinians who live in occupied territories are mostly living in unbearable circumstances due to a lack of their basic human needs being met [4]. Palestinians should have their freedom to travel, and have easy access to all their basic needs as human beings [20].

The mental health system needs to move toward vertical and horizontal integration of services. The style of care should move toward multidisciplinary working based on non-hierarchical mental health teams. Patients and families should be at the heart of interdisciplinary care. Mental health care should be integrated into the rest of primary and secondary health care such as public hospitals [20]. According to the WHO [29] strategic directions for 2011–2015 it is important to focus on strengthening nursing and midwifery services. Marie [33] suggested that there is an increasing need for more and better quality studies in the Palestinian mental health field, and from nursing researchers’ perspectives specifically given the importance of nurses for the development of health systems. There is a priority for more accurate and representative surveys about the mental health of Palestinians. There is a need for more cooperation between researchers in order to enhance the accumulation of knowledge, especially in the area of screening. Mental health nurses have reported that they are neglected and unable to provide the quality of care they wish. There is a significant need to effectively engage nurses in managing available resources and share effectively in the development and administration of policies. This will have a promise to improve meeting users’ needs and develop mental health services [43]. Due to the lack of psychiatrists in the field, non-medical professionals such as mental health nurses or social workers can be empowered in order to share effectively in offering multidisplinary care plans for clients. There are many duties that the multidisciplinary team can do instead of psychiatrists such as teaching [20], referrals and assessment [49]. For example, we suggest empowering and training mental health nurses in order to prescribe specific monthly medications independently as in the UK [50]. Cooperation and connections between mental health professionals in Palestine and other countries will also help to develop and increase the capacity of Palestinian mental health teams [20].

Finally, there is a significant need to minimise the challenges and develop resiliency or capacity among civilians. Civilians also need to focus on their own available resilience resources and protective factors in order to survive. The outcomes might help decision makers to focus on how to develop resiliency, support the Palestinian population and mental health professionals. Resilient teams can inspire service users, and lead and play a crucial role in developing mental health services.

## Limitations

This literature review has discussed mental health needs and services in Palestine. Palestine has been described as “uncharted territories’’ due to a lack of accurate data, resources or records [51]. It is a state that is seeking independence with scarce resources, therefore health research is underdeveloped and there is a significant lack of health research infrastructure and funding [52]. As a result, there is a lack of detailed data or national surveys relating to the mental health field [20]. In the absence of a mature programme of research, this review has had to rely instead on formal reports and small-scale, fragmented studies completed by academics or by nongovernmental organisations with little or no funding. However, also important to note is that nearly all of the reports used were produced by reliable international and national organisations such as WHO, UN, Amnesty International and MOH.
